# Phosphorus Modified Cardanol: A Greener Route to Reduce VolaTile Organic Compounds and Impart Flame Retardant Properties to Alkyd Resin Coatings

**DOI:** 10.3390/molecules27154880

**Published:** 2022-07-30

**Authors:** Maxinne Denis, Damien Le Borgne, Rodolphe Sonnier, Sylvain Caillol, Cédric Totee, Claire Negrell

**Affiliations:** 1 Institute Charles Gerhardt Montpellier (ICGM), Université de Montpellier, Recherche Scientifique (CNRS), Ecole Nationale Supérieure de Chimie de Montpellier (ENSCM), 34000 Montpellier, France; maxinne.denis@enscm.fr (M.D.); sylvain.caillol@enscm.fr (S.C.); cedric.totee@enscm.fr (C.T.); 2 Lixol, Groupe Berkem, 20 Rue Jean Duvert, 33290 Blanquefort, France; damien.leborgne@berkem.com; 3 Polymers Composites and Hybrids (PCH), IMT Mines Ales, 30100 Ales, France; rodolphe.sonnier@mines-ales.fr

**Keywords:** alkyd resins, VOC, reactive diluent, cardanol, phosphonate, phosphate, flame retardant

## Abstract

Novel phosphorylated cardanol molecules based on phosphonate (PO_3_CR) and phosphate (PO_4_CR) functions were synthetized. Those molecules have two main actions which are described in this article: the reduction in volatile organic compounds (VOC) and the development of flame retardant (FR) properties conferred on alkyd resins used as coatings for wood specimen. Phosphorylated cardanol compounds have been successfully grafted by covalent bonds to alkyd resins thanks to an auto-oxidative reaction. The impact of the introduction of PO_3_CR and PO_4_CR on the film properties such as drying time and flexibility has been studied and the thermal and flame retardant properties through differential scanning calorimeter, thermogravimetric analysis and pyrolysis-combustion flow calorimeter. These studies underscored an increase in the thermal stability and FR properties of the alkyd resins. In the cone calorimeter test, the lowest pHRR was obtained with 3 wt% P of phosphate-cardanol and exhibited a value of 170 KW.m^−2^, which represented a decrease of almost 46% compared to the PO_x_CR-free alkyd resins. Moreover, a difference in the mode of action between phosphonate and phosphate compounds has been highlighted. The most effective coating which combined excellent FR properties and good coating properties has been obtained with 2 wt% P of phosphate-cardanol. Indeed, the film properties were closed to the PO_x_CR-free alkyd resin and the pHRR decreased by 41% compared to the reference alkyd resin.

## 1. Introduction

Since their first synthesis in the mid-1920s, alkyd resins have been widely used as binders in paints and varnishes for coating applications [1,2,3,4]. Alkyd resins are polyesters obtained by a polycondensation reaction between fatty acids and vegetable oils, anhydrides and acids, and polyols [5,6,7]. Alkyd resins present the major advantage of being composed mainly of biobased raw materials. Nevertheless, to allow application by reducing the viscosity, traditional alkyd coating formulations are composed of 20 to 60 wt% of solvent. Those solvents are usually hydrocarbons such as xylene or white spirit, derived from fossil resources [8,9]. Those are volatile organic compounds (VOC), released into the atmosphere that may lead to environmental and human health concerns [10,11]. Hence, their use is limited by European regulations [1,7,12]. Therefore, for several decades, research has focused on the development of water-based alkyd coatings through the use of emulsifiers [13]. Despite great progress, waterborne alkyd coatings suffer from poor scrub resistance, low corrosion resistance, an undesirable yellowing effect and the necessity for the use of biocide [7,13,14]. In order to overcome these limitations, inorganic or organic fillers have to be incorporated into water-based alkyd coatings [15]. Nevertheless, these fillers could be expensive and modify the mechanical properties. An effective approach to reducing the VOC content of alkyd coating formulations and maintaining traditional alkyd performances is the use of reactive diluents to replace a part of the volatile solvent. Reactive diluents are chemical compounds that reduce the viscosity of the formulation while reacting with the polymer during the curing and therefore reducing VOC content [16,17]. Hence, such reactive diluents need a low viscosity, low volatility, great compatibility to promote solubility and the capacity to form a 3D network with alkyd resins during the drying process by auto-oxidative reactions [18,19,20,21].

Nowadays, the use of industrial wastes or by-products is one of the environmental challenges faced by many industries and universities over the past few years [6]. Cashew nut shell liquid (CNSL), is a non-edible by-product of the cashew nut industry, directly extracted from the cashew nut shell [22]. With an annual production approaching one million tons, CNSL is a major and economical renewable resource [23,24]. Cardanol is readily obtained from CNSL, composed of a phenol group that can provide corrosion resistance, and of an unsaturated fatty chain compatible with alkyd resins and able to achieve auto-oxidative reaction during the curing process. Wang et al. studied the advantages of cardanol (CO), cardanol methacrylate (MACO) and triethoxysilane-functionalized cardanol (TSCO) as reactive diluents for alkyd coatings [25]. They have demonstrated that the viscosity of the alkyd resins decreased with the growing amount of CO, MACO and TSCO. In a second study, the authors demonstrated that the introduction of 5 wt% MACO or TSCO can reduce the amount of xylene up to 9 wt% while maintaining the viscosity of the alkyd coating formulations [13].

Moreover, like all polymers, alkyd resins suffer from their flammability because of their high contents of hydrogen and carbon elements. The new efficient generation of environmentally friendly flame retardants (FR) combines phosphorus-containing groups and biobased compounds [26]. The association of cardanol with phosphonate or phosphate functions could provide a double solution in terms of diluent and FR properties. One way to overcome the flammability is the addition of flame retardant (FR) in the polymer matrix. Pillai et al. have synthetized a flame retardant through the modification of the cardanol hydroxyl group with phosphoric acid [24]. This phosphorylated cardanol exhibited high thermal stability. Ecochard et al. have investigated the flame retardancy of phosphoryl chloride (POCl_3_) modified cardanol in epoxy resins [27]. These resins exhibited a lower peak of heat release rate (pHRR) and total heat released (THR) in cone calorimeter analysis as well as an increase in char residue which indicated good flame retardancy properties. Phalak et al. have synthetized an acrylated cardanol diphenyl phosphate with flame retardant properties [28]. The UV-cured coatings exhibited higher char residue and enhanced LOI value and UL-94 rating. Mestry et al. have demonstrated the increase in LOI value and char residue for polyurethane coatings based on cardanol modified with phenylphosphonic dichloride [29].

**Scheme 1:**
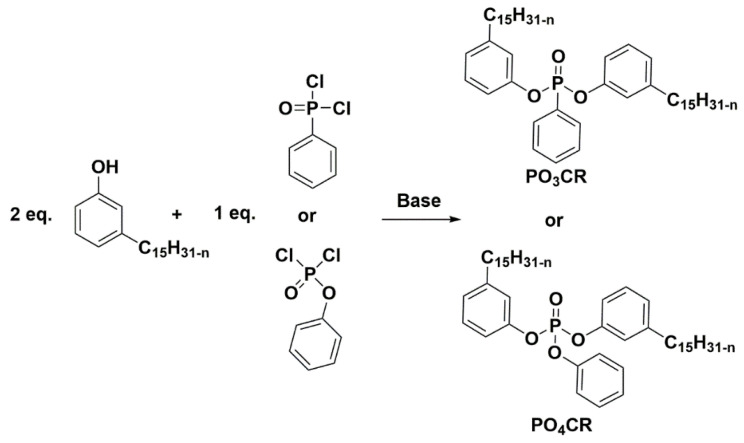
General synthesis of the phosphonate- or phosphate-cardanol as a reactive diluent (PO_3_CR and PO_4_CR).

Furthermore, to the best of our knowledge, a cardanol-based FR reactive diluent has never been reported in the literature. Hence, this article studies the modification of cardanol with phosphorus-containing compounds (Scheme 1) in order to synthetize novel reactive diluents with flame retardant properties. The phosphorylated cardanol reactive (PO_x_CR) diluents were introduced in alkyd resin formulations to reduce the amount of VOC. Molecular structures of PO_x_CR diluents were characterized by nuclear magnetic resonance (NMR), Fourier transform infrared (FT-IR) and alkyd resins by size exclusion chromatography (SEC). The viscosity of the alkyd resins was determined by rheological analysis. The flame retardancy properties were investigated by thermogravimetric analysis (TGA), pyrolysis combustion flow calorimeter analysis (PCFC) and cone calorimeter. Char formation and heat release rate were discussed in function of phosphonate and phosphate molecules. Furthermore, the film properties of alkyd resins were also studied through characterizations such as gloss, hardness, chemical resistance and drying time.

## 2. Materials and Methods

### 2.1. Materials

Tall oil fatty acid (TOFA), glycerol, phthalic anhydride, benzoic acid, a mix of driers (a solution containing cobalt octoate, calcium octoate and zirconium octoate) were kindly supplied by Lixol (Groupe Berkem, Blanquefort, France). Cardanol NX-2026 was kindly provided by Cardolite. Phenylphosphonic dichloride, phenyl dichlorophosphate, triethylamine (TEA) and xylene were purchased from Sigma Aldrich. Sodium chloride (NaCl), sodium bicarbonate (NaHCO3), hydrochloric acid (HCl), magnesium sulfate (MgSO4), dichloromethane (DCM) and tetrahydrofuran (THF) were purchased from VWR International S.A.S (Fontenay-sous-Bois, France). The NMR solvents used were CDCl3 and THF-d8 from Eurisotop. All reagents were used as received.

### 2.2. Methods

#### 2.2.1. Nuclear Magnetic Resonance

NMR samples were prepared with CDCl_3_ as a solvent and the analyses were performed using a Bruker Avance I 400 MHz spectrometer at 25 °C. The structure of monomers was determined by hydrogen nuclear magnetic resonance (^1^H NMR), and phosphorus nuclear magnetic resonance (^31^P NMR). The ^1^H NMR spectra were recorded at 8 kHz for spectral width, 3 kHz for transmitter frequency offset, 4 s for acquisition time and 8 scans were performed. Quantitivity conditions were obtained with a 30° pulse (3 µs) and a relaxation delay at 1 s. The ^31^P NMR spectra were recorded at 64 kHz for spectral width, 8000 Hz for transmitter frequency offset, 1 s for acquisition time and 4 scans were performed. Quantitivity conditions were obtained with a 30° pulse (2.7 µs) and a relaxation delay at 20 s. External references were trimethylsilane (TMS) for ^1^H and phosphoric acid (H_3_PO_4_) for ^31^P NMR. Shifts were given in ppm. Furthermore, to confirm the crosslinking between the alkyd resins and phosphorylated cardanol monomers high-resolution magic angle spinning (HRMAS) NMR experiments have been investigated. HRMAS NMR experiments were carried out on a Varian VNMRS 600 MHz spectrometer equipped with a wide bore magnet (B0 = 14.1 T). Prior to ^1^H HRMAS NMR experiments, cured alkyd resins were ground thanks to a cryogenic grinder and the powders were injected into a 4 mm quartz zirconia HRMAS rotor. Then, THF-d_8_ was injected as solvent and experiments were performed at 20 °C, 9.6 kHz for spectral width, 1200 Hz for transmitter frequency offset, 1.7 s for acquisition time and 8 scans. Quantitivity conditions were obtained with a 30° pulse (3.9 µs) and a relaxation delay at 1 s.

#### 2.2.2. Acid Value (*AV*)

Acid values were determined according to ISO 2114:2000 standard. It corresponds to the mass of potassium hydroxide (KOH) in milligrams that are required to neutralize one gram of alkyd resin mass. Approximatively, 1 g of the reaction mixture was withdrawn and solubilized in a neutralizing solution (80/20 xylene/ethanol). The solution was then titrated with a KOH solution at 0.1 mol.mL^−1^. Thanks to the following Equation (1), the acid value was calculated.

(1) $$AV=\frac{V_{KOH}\times M_{KOH}\times C_{KOH}}{m\times N.V.C.}$$

*AV*: Acid value; *V_KOH_*: volume of *KOH* solution introduced to neutralize the alkyd resin (mL); *M_KOH_*: molecular weight of *KOH* (g.mol^−1^); *C_KOH_*: concentration of KOH (mol.mL^−1^); *m*: mass of alkyd resin withdraw (g); *N*.*V*.*C*.: Non Volatile Content of the sampled resin.

#### 2.2.3. Rheological Analysis

Rheological analyses were performed on a ThermoScientific Haake Mars 60 rheometer equipped with a 35-mm-cone-plate geometry. The analyses were performed at 20 °C with a shear rate of 10 s^−1^.

#### 2.2.4. Size-Exclusion Chromatography (SEC)

Molecular weights of alkyd resins were determined by size-exclusion chromatography (SEC). SEC was recorded using a triple-detection GPC from Agilent Technologies with its corresponding Agilent software, dedicated to multidetector GPC calculation. The system used two PL1113-6300 ResiPore 300 × 7.5 mm columns with THF as the eluent with a flow rate of 1 mL·min^−1^. The detector was a 390-LC PL0390-0601 refractive index detector. The entire SEC-HPLC system was thermostated at 35 °C. Polymethylmethacrylate (PMMA) standards were used for calibration between 540 and 2,210,000 g·mol^−1^. The typical sample concentration was 15 mg·mL^−1^.

#### 2.2.5. Gel Content (*GC*)

The resins were mixed with 5 wt% of a mix of driers (cobalt octoate, calcium octoate and zirconium octoate) to crosslink them with the phosphorus reactive diluent. Then, three samples from the same alkyd resin films, of around 20 mg each, were separately immersed in THF for 24 h. The three samples were then dried in a ventilated oven at 70 °C for 24 h. The gel content (*GC*) was calculated using Equation (2), where m_2_ is the mass of the dried material and m_1_ is the initial mass. The reported gel contents are the average values of the three samples.

(2) $$GC=\frac{m_{2}}{m_{1}}\times 100$$

#### 2.2.6. Differential Scanning Calorimetry

Differential scanning calorimetry (DSC) analyses were carried out using a Netzsch DSC200F3 calorimeter. The calibration was performed using adamantane, biphenyl, indium, tin, bismuth and zinc standards. Nitrogen was used as the purge gas. The thermal properties were analyzed at 20 °C/min between −100 and 100 °C to observe the glass transition temperature.

#### 2.2.7. Thermogravimetric Analysis (TGA)

Thermogravimetric analyses (TGA) of the cured alkyd resins were carried out to determine the thermal stability and were performed on a Netzsch TG 209F1 apparatus under 40 mL.min^−1^ nitrogen flow. The protective gas used was nitrogen with a 20 mL.min^−1^ flow. Approximately 10–12 mg of sample was placed in analumina crucible and heated from room temperature to 850 °C with a 20 °C.min^−1^ heating rate.

#### 2.2.8. Pyrolysis Combustion Flow Calorimeter (PCFC)

The flammability of resins was analyzed using a pyrolysis combustion flow calorimeter (PCFC). About 3–4 mg were placed in the pyrolyzer, undergoing an increase in temperature from 100 °C to 750 °C at a rate of 1 °C.s^−1^ under a nitrogen flow. Pyrolytic gases were sent to a combustor heated at 900 °C under airflow (N_2_/O_2_ = 80/20). At this temperature and with 20% of oxygen, combustion was considered to be complete. Heat release rate (HRR) was determined according to oxygen depletion (Huggett’s relation) as in the cone calorimeter test. PCFC analyses correspond to anaerobic pyrolysis followed by high-temperature oxidation of decomposition products (complete combustion) [30]. All samples were tested in triplicate.

#### 2.2.9. Cone Calorimeter

Cone calorimeter tests were carried out to investigate the fire behavior of resins used as a coating on wood specimens. The alkyd resins were applied on a pine wood sample (100 × 100 × 25 mm) to obtain a coating of 0.01 g.cm^−2^. The samples were placed at 2.5 cm below a conic heater and isolated by rock wool. The samples were exposed to a 35 kW.m^−2^ heat flux in well-ventilated conditions (air rate 24 L.s^−1^) in the presence of a spark igniter to force the ignition. Heat release rate (HRR) was determined by oxygen depletion according to the Huggett principle (1 kg of consumed oxygen corresponds to 13.1 MJ of heat released) [31]. The Peak of Heat Release Rate (pHRR) is the maximal value of the heat release rate. The total heat released (THR) was obtained by integration of HRR curves. All samples were tested in triplicate.

#### 2.2.10. Film Properties

A total of 100 µm of wet films were applied with a film applicator and dried at 25 °C under a relative humidity of 30%. The dry film thickness was 60 µm in all cases. Adhesion was measured using a cross-cut tester 1 mm BYK according to ISO 2409:2020 standard. The hardness (Persoz hardness) was determined according to ISO 1522:2006 standard with a TQC SP0500 pendulum hardness tester. Gloss was measured based on ISO 2813:2014 standard and the measurements were performed on substrates at 60° and 20° using a TQC Polygloss. The values for the hardness and the gloss measurements were determined at day +10 (D + 10) after the application of the film. The drying time of the resins was considered as the time required to obtain a tack-free film. The chemical resistance of the alkyd resins was studied in water (H_2_O), hydrochloric acid (HCl), sodium hydroxide (NaOH) and sodium chloride (NaCl) [30]. Approximatively 30 ± 1 mg of the cured alkyd resins were immersed inside each solvent over six hours at room temperature and stirred at 200 rpm. Then, they were dried in an oven at 70 °C for 12 h. The residual mass percentage (wt% residue) was determined using Equation (3). The analyses were made in triplicate for each sample and the reported residual mass percentages are average values of the three samples.

(3) $$wt\%\;residue=\frac{m_{2}}{m_{1}}\times 100$$

*m*_1_: mass of the alkyd resin sample before being immersed in solvent; *m*_2_: mass of the alkyd resin sample after being immersed in a solvent and dried in oven.

#### 2.2.11. Synthesis of Phosphonate Cardanol Diluent (PO_3_RC)

**Figure 1:**
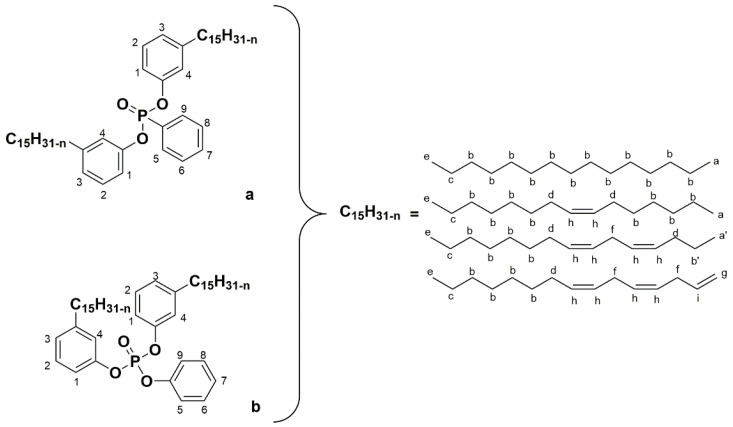
Structure of (**a**) phosphonate- and (**b**) phosphate-cardanol.

A solution of 100 g cardanol NX-2026 (0.333 mol, 2 equiv.) and 37.03 g of triethylamine (0.366 mol, 2.2 equiv.) in 500 mL of dichloromethane was purged with nitrogen for 20 min and cooled down to 0 °C. Then, 32.5 g of phenylphosphonic dichloride (0.166 mol, 1 equiv.) was added dropwise over 1 h with a dropping funnel. The mixture was stirred for an additional 30 min at 0 °C and then allowed to warm up to room temperature. The mixture was stirred overnight. When the reaction was completed, the mixture was washed three times with 300 mL of HCl 1M solution, a saturated aqueous solution of NaHCO_3_ and then a saturated aqueous solution of NaCl. The organic layer was dried with anhydrous MgSO_4_ and distilled under reduced pressure (10 mbar) at 30 °C to give 114.7 g of phosphonate-cardanol as a pale-yellow oil (95% yield). The phosphonate-cardanol exhibited a phosphorus percentage of 4.3%. The structure of phosphonate-cardanol is presented in Figure 1a.

##### ^31^P NMR (161.6 MHz, CDCl_3_, ppm): δ: 11.5

^1^H NMR (400 MHz, CDCl_3_, ppm): δ: 7.99 (m, 2H, aromatic protons 5 and 9), 7.59 (dt, 1H, aromatic protons 7), 7.50 (m, 2H aromatic protons 6 and 8), 7.19 (t, 2H, aromatic protons 2), 6.99 (m, 6H, aromatic protons 1, 3, and 4), 5.80–5.90 (m, 1H, CH of C_15_ chain, proton i), 5.34–5.50 (m, CH of C_15_ chain, internal double bond protons h), 4.98–5.11 (m, 2H, CH_2_ of C_15_ chain, protons of the terminal double bond g), 2.82 (m, 2H, CH_2_ of C_15_ chain, protons f), 2.61 (t, 2H, CH_2_ of C_15_ chain, protons e), 2.00–2.15 (m, 2H, CH_2_ of C_15_ chain, protons d), 1.64 (m, 2H, CH_2_ of C_15_ chain, protons c), 1.20–1.50 (m, CH_2_ of C_15_ chain, protons b, b′), 0.92 (m, 3H, CH_3_ of C_15_ chain, terminal –CH_3_, protons a, a′).

#### 2.2.12. Synthesis of Phosphate Cardanol Diluent (PO_4_RC)

A solution of 100 g cardanol NX-2026 (0.333 mol, 2 equiv.) and 37.03 g triethylamine (0.366 mol, 2.2 equiv.) in 500 mL of dichloromethane was purged with nitrogen for 20 min and cooled down to 0 °C. Then, 35.1 g of phenyl dichlorophosphate (0.166, 1 equiv.) was added dropwise over 1 h with a dropping funnel. The mixture was stirred for an additional 30 min at 0 °C and then allowed to warm up to room temperature. The mixture was stirred overnight. When the reaction was completed, the mixture was washed three times with 300 mL of HCl 1M solution, a saturated aqueous solution of NaHCO_3_ and then a saturated aqueous solution of NaCl. The organic layer was dried with anhydrous MgSO_4_ and distilled under reduced pressure (10 mbar) at 30 °C to give 115.9 g of phosphonate cardanol as a pale-yellow oil (94% yield). The phosphate-cardanol exhibited a phosphorus percentage of 4.2%. The structure of phosphate-cardanol is presented in Figure 1b.

##### ^31^P NMR (161.6 MHz, CDCl_3_, ppm): δ: −17.7

^1^H NMR (400 MHz, CDCl_3_, ppm): δ: 7.37 (m, 2H, aromatic protons 5 and 9), 7.24–7.28 (m, 3H, aromatic protons 6, 7 and 8), 7.19–7.24 (m, 2H, aromatic proton 2), 7.05 (m, 6H, aromatic protons 1, 3, and 4), 5.80–5.90 (m, 1H, CH of C_15_ chain, proton i), 5.34–5.50 (m, CH of C_15_ chain, internal double bond protons h), 4.98–5.11 (m, 2H, CH_2_ of C_15_ chain, protons of the terminal double bond g), 2.82 (m, 2H, CH_2_ of C_15_ chain, protons f), 2.61 (t, 2H, CH_2_ of C_15_ chain, protons e), 2.00–2.15 (m, 2H, CH_2_ of C_15_ chain, protons d), 1.64 (m, 2H, CH_2_ of C_15_ chain, protons c), 1.20–1.50 (m, CH_2_ of C_15_ chain, protons b, b′), 0.92 (m, 3H, CH_3_ of C_15_ chain, terminal –CH_3_, protons a, a′).

#### 2.2.13. Synthesis of Tall Oil Fatty Acids (TOFA) Alkyd Resin

A short oil TOFA alkyd resin was prepared by a solvent process using TOFA, glycerol, phthalic anhydride and benzoic acid. TOFA and glycerol were first added in a five-necked reactor flask equipped with a mechanical stirrer, a Dean Stark, a thermometer and a nitrogen inlet. Then, when the mixture reached 200 °C, phthalic anhydride and benzoic acid were added. The reaction mixture was heated to 220 °C using xylene as an azeotropic solvent to remove water. The reaction was continued until an acid value of 14 mg of KOH/g of resin was reached.

#### 2.2.14. Preparation of the Mixture Alkyd Resin—Reactive Diluent

Different ratios of PO_x_CR diluents (with x = 3 or 4) were added to obtain alkyd resins with 0 wt%, 1 wt%, 2 wt% and 3 wt% of phosphorus. The different formulations of alkyd resins with phosphorylated cardanol reactive diluents are presented in Table 1. 

## 3. Results and Discussion

### 3.1. Synthesis of Phosphorus Cardanol Reactive (PO_x_CR) Diluent Containing TOFA Alkyd Resin

**Scheme 2:**
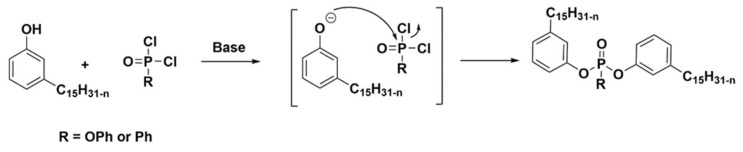
Mechanism of Williamson reaction with phosphorus dichloride compounds.

The synthesis of PO_x_CR diluents (with x = 3 or 4) is represented in Scheme 2. The derivative Williamson reaction with phosphorus-containing compounds has been widely investigated in several articles [27,32,33,34]. Phosphorus chloride and cardanol were added in a ratio of 1:2 and stirred overnight at room temperature. This Williamson reaction allowed the formation of the P-O bond through an SN_2_ mechanism.

**Figure 2:**
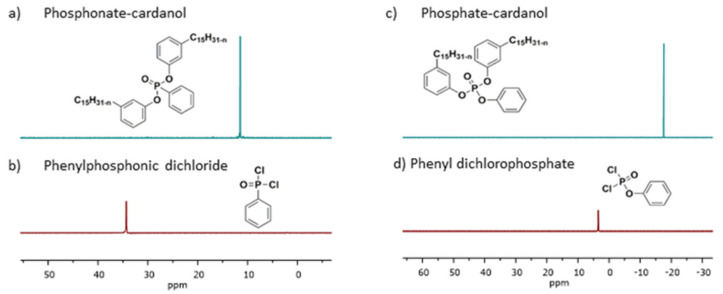
^31^P NMR spectra of (**a**) phosphonate- and (**b**) phosphate-cardanol compounds and their (**c**) and (**d**) initial phosphorylated reagents.

Phosphorus-containing cardanol molecules were characterized by ^31^P and ^1^H NMR (Figures S1 and S2) spectroscopy. The ^31^P NMR spectra in CDCl_3_ are presented in Figure 2. The disappearance of the signal at 34.4 ppm for phenylphosphonic dichloride in the ^31^P NMR spectra and the appearance of the chemical shift at 11.5 ppm confirmed the formation of phosphonate-cardanol (Figure 2a,b). Likewise, in the ^31^P spectrum, the disappearance of the chemical shift at 3.2 ppm for phenyl dichlorophosphate and the appearance of the chemical shift at −17.7 ppm demonstrated the formation of phosphate cardanol molecule through P-O bonds (Figure 2c,d).

**Scheme 3:**
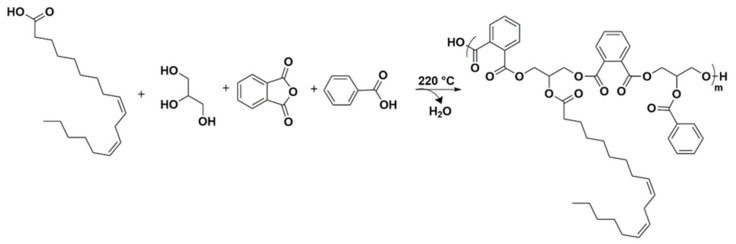
Synthesis of TOFA alkyd resin.

The alkyd resins were synthetized through a fatty acid process with the presence of xylene as an azeotropic solvent. The presence of a low amount of xylene (9 wt%) is necessary to allow the progress of the esterification reaction. The synthesis of tall oil fatty acid (TOFA) alkyd resin is presented in Scheme 3. The resins have been designed to present the same weight percentage of oil in their composition (also named oil length) of 38 wt%, which is why they can be classified as short oil length resins.

Size exclusion chromatography (SEC) allowed to determine the weight average molecular weight Mw of the resins. The results (PMMA equivalent) exhibited Mw values at 200 000 g.mol^−1^. High molecular weight is characteristic of short oil alkyd resins since the molecules are more compact, rigid and have less fatty acid side chain in the polymer linkages [35].

A minimal amount of 9 wt% of xylene was required to obtain a homogenous solution. Nevertheless, this study focused on the maximal reduction in solvent content to reduce the VOC of the final alkyd resins. Thereby, the PO_x_CR diluents were introduced with different phosphorus percentages in the resins.

The amount of xylene was adjusted in order to reach a viscosity of 5.3 ± 0.2 Pa.s for all the resins, which is the viscosity of the reference alkyd resin without reactive diluent (Table 1).

Then, the non-volatile content (NVC) was determined thanks to a desiccant balance. As expected, the growing amount of PO_x_CR diluent introduced in alkyd resins allowed to reduce the amount of solvent to reach the same viscosity at ±0.2 Pa.s for all the alkyd resins. The increased amount of PO_x_CR diluent introduced, decreased the quantity of alkyd resin, which also impact the viscosity diminution. The introduction of 3 wt% of phosphorus (3 wt% P) with PO_3_CR and PO_4_CR diluent allowed the reduction of the amount of solvent by 30 wt% compared to the reference alkyd resin. Thereby, the introduction of reactive diluent allowed a sharp reduction in the volatile compounds.

Then, in order to have comparable results with the alkyd resin without reactive diluent for the following analyses, the viscosities of all the resins were also compared with the same non-volatile content of 60 wt%, as presented in Table 2. First, the introduction of 1 wt% P, which represents 16 wt% and 17 wt% of PO_3_CR and PO_4_CR diluents, reduced the viscosity by 68% compared to the reference alkyd resin. With a phosphorus percentage increased from 2 wt% P to 3 wt% P, the viscosity decreased from 0.17 to 0.03 Pa.s, as presented by the asymptote curve (Figure S11). As alkyd resins are used for a wide range of paint applications, each application requires a specific viscosity. Hence, these reactive diluents offer the possibility to modify the viscosity and reduce the VOC content.

Due to the fixed value of the volatile content, the dry films obtained exhibited the same final thickness of 60 µm which allows the comparison of the film characteristics.

During the curing of the resins, the PO_x_CR diluents were crosslinked to the alkyd resins thanks to the introduction of 5 wt% of a mix of driers (cobalt octoate, calcium octoate and zirconium octoate), a catalyst of autoxidation process. Indeed, cardanol and alkyd resins are both composed of unsaturated fatty acids which allow oxygen-curing in air [36]. The curing process occurs in three steps: peroxidation, peroxide decomposition and cross-linking reaction that occurs by a free-radical mechanism. Firstly, the oxygen penetrates the film allowing the oxidation of fatty acids and promoting the formation of hydroperoxides. As soon as they are formed, the hydroperoxides dissociate into free radicals (ROO^●^ and RO^●^) which react through radical-radical combinations to form covalent carbon-carbon, ether, and peroxide bonds. To confirm the presence of a crosslinked network, different analyses were performed such as the determination of the gel content (*GC*) and the investigation of the disappearance of the chemical shift of unsaturations (C=C) by ^1^H HRMAS NMR. The *GC* was defined as the mass of insoluble material after being exposed for 24 h in THF solvent. Indeed, an insoluble 3D network is formed when the alkyd resin is cured. For all the alkyd resins, the *GC* was higher than 91% confirming the crosslinking (Table 2). The *GC* of the reference alkyd resin exhibited the highest value with 95%. Nevertheless, the *GC* slightly decreased with the amount of phosphorylated cardanol. This may be attributed to the percentage of saturated chains of cardanol which accounted for 5–8% of the alkyl chains [22]. Indeed, depending on the amount of phosphorylated cardanol introduced, the percentage of saturated chains oscillated between 1 and 5 wt% of the total mass of the alkyd resins. Those values were in accordance with the *GC* results. Moreover, all the resins exhibited a very close *GC*, allowing us to conclude that phosphorylated cardanol created covalent bonds with the polymer matrix and did not deteriorate the crosslinking density. ^1^H HRMAS experiments were also performed to confirm the crosslinking between alkyd resin and phosphorylated cardanol. In all the HRMAS NMR spectra (Figures S8–S10) of the phosphorylated cardanol-modified alkyd resins, the peaks of the protons of the terminal double bond (i and g Figure 1) disappeared. This result was expected because the protons of the terminal double bonds are more reactive than the protons of internal double bonds in the alkyl chains. Moreover, it showed that the phosphorylated cardanol has been cross-linked with the alkyd resin. ^1^H NMR spectra of the alkyd resins before curing have been performed and are presented in Figures S3–S6. Then, ^1^H HRMAS NMR spectra of all the alkyd resins have been achieved and are shown in Figures S7–S10. Nevertheless, only the reference alkyd resin and phosphate cardanol alkyd resins exhibited HRMAS NMR spectra with good resolutions. For this reason, analyses have been performed only on those resins. The peak at 5.4–5.7 ppm corresponded to the protons of the internal double bonds. The decrease of the signal of these protons by 50% for the reference alkyd resin and completely for the phosphorylated cardanol-modified alkyd resins is consistent with the results obtained for the *GC* and confirmed the crosslinking between phosphorylated cardanol and alkyd resins. These results allowed us to consider similar results with phosphonate-cardanol alkyd resins since the change of oxidation degree of the phosphorus function had no influence on the grafting of double bonds during the alkyd resin drying.

Differential scanning calorimetry (DSC) analyses were carried out to determine the glass transition temperature (Tg) of curing films and are summarized in Table 2. The curves are presented in Figure S12. Indeed, the flexible alkyl chain and bulky phenyl groups of PO_x_CR diluents may impact the physical properties of the resins compared to the use of xylene that is theoretically removed during the drying step. The PO_x_CR-free resin exhibited a Tg value of 40 °C while the introduction of PO_x_CR diluent (causing the diminution of the alkyd resin content) reduced the Tg values of modified alkyd resins. Indeed, for alkyd resins containing phosphonate-cardanol, Tg values ranged from 27 °C for the alkyd resin with 1 wt% P to −11 °C for the alkyd resin with 3 wt% P. This evolution was mainly due to the introduction of alkyl chains provided by PO_x_CR diluent which exhibited a low molecular weight and improved flexibility thanks to the alkyl chain of cardanol. Moreover, the decrease in polymer content with the increased amount of PO_x_CR diluent also reduced the Tg. These results showed that the oxidation state of phosphorus did not have an influence on Tg values. The Tg of 2 wt% P alkyd resins were −3 °C for both PO_3_CR and PO_4_CR. Depending on the Tg values, the application of alkyd resins could be different. Indeed, alkyd resins with negative Tg would rather be used for surfaces that required flexible coatings such as wood. However, high Tg would instead be used for a surface that required a hard coating such as metal.

### 3.2. Thermal and Flame Retardant Properties

**Figure 3:**
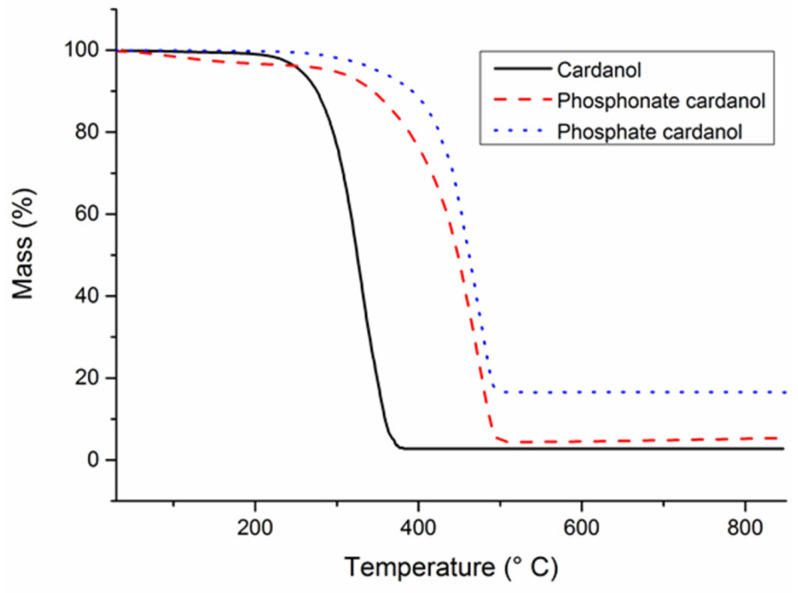
TGA thermograms of cardanol, phosphonate-cardanol and phosphate-cardanol.

Thermal and flame retardancy properties were investigated in this part to highlight the influence of the PO_x_CR diluents in alkyd resins. Firstly, the thermal decomposition of PO_3_CR and PO_4_CR was studied and compared to the thermal decomposition of cardanol, as presented in Figure 3. Phosphonate and phosphate functions improved the thermal stability of cardanol. Indeed, the temperature at 50% weight loss (T_d,50wt%_) increased by 131 and 139 °C for phosphonate-cardanol (448 °C) and phosphate-cardanol (462 °C) compared to the neat cardanol (350 °C). Moreover, the char yield at 850 °C was also increased for the cardanol grafted with phosphorus compounds. Indeed, the residual yields were 2.6%, 5.4% and 16.5%, respectively, for cardanol, phosphonate-cardanol and phosphate-cardanol. Those results were expected as phosphonate and phosphate compounds act in the condensed phase by promoting char formation. Indeed, rearrangement and Diels–Alder reactions led to the formation of a carbonaceous layer [37]. However, phosphonate compounds are much less efficient than phosphate compounds to promote char.

**Figure 4:**
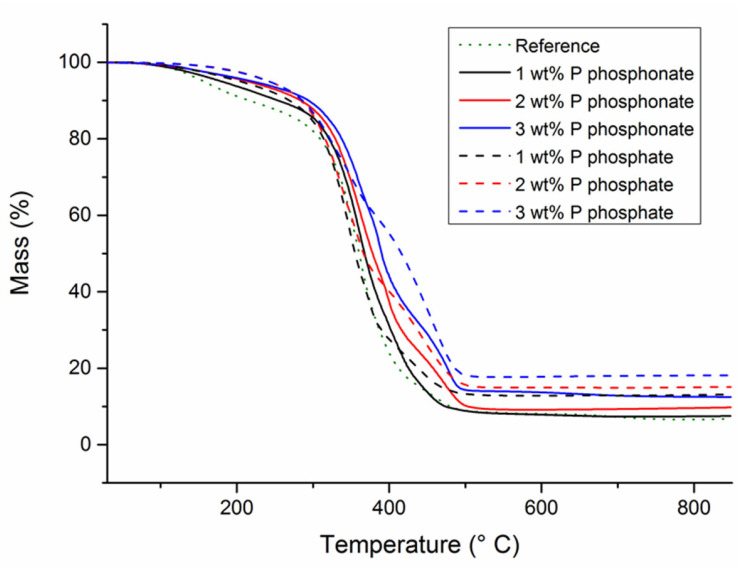
TGA thermograms of reference alkyd resin and phosphorylated cardanol modified alkyd resins.

**Scheme 4:**
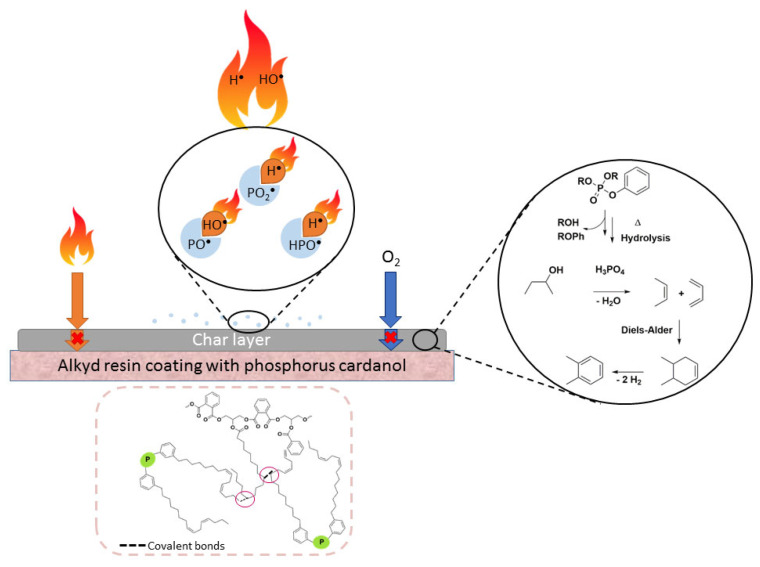
Schematic illustration of possible flame retardant mechanism.

The thermal properties of all alkyd resin films were investigated by thermogravimetric analyses (TGA) under nitrogen flow. Figure 4 shows the thermograms of the alkyd resins, and with, respectively, 1 wt%, 2 wt%, and 3 wt% of phosphorus brought by PO_x_CR diluent. Thermal properties such as the decomposition temperature at 5% weight loss, the temperature at 50% weight loss, and the residue yield at 850 °C, are summarized in Table 3. Thermal stability increased with the addition of PO_3_CR or PO_4_CR. Firstly, the temperature at 5% weight (T_d,5wt%_) increased from 140 °C for the alkyd resin without PO_x_CR diluent to 180 °C and 204 °C for the resins with, respectively, 1 wt% P of PO_3_CR and 1 wt% P of PO_4_CR. Moreover, the growing amount of PO_x_CR introduced in alkyd resins also increased the T_d,5wt%_. For example, the T_d,5wt%_ increased by 41 °C as the amount of phosphonate-cardanol increased from 1 wt% P to 3 wt% P. Considering the temperature at 50% weight loss (T_d,50wt%_) the thermal stability was higher with the increasing the amount of PO_x_CR. Indeed, T_d,50wt%_ varied from 350 °C for the reference resin to 390 °C and 416 °C for the resins with 3 wt% P of phosphonate-cardanol and 3 wt% P of phosphate-cardanol, respectively. As expected, the thermal stability of both PO_3_CR and PO_4_CR alkyd resins is similar but slightly higher for phosphate in good agreement with the stability of PO_4_CR. During combustion, PO_3_CR and PO_4_CR may generate free radicals such as PO^●^, HPO^●^, PO_2_^●^. Those radicals can quench the active radicals generated by the flame (H^●^ and OH^●^) and then, reduce the combustion in the gaseous phase, as presented in Scheme 4 [38,39]. Nevertheless, no evidence of such mode-of-action can be provided in the present paper. Indeed, flame inhibition may be assessed by calculating the combustion efficiency in a cone calorimeter (from the comparison between the heat of complete combustion and the effective heat of combustion). Unfortunately, while the alkyd resins are used as a thin coating on woods, effective heat of combustion is mainly driven by wood combustion. Therefore, flame inhibition may be only suggested. The residue yield was impacted by the oxidation degree of the phosphorus, as well as the amount of phosphorus introduced in alkyd resins. The reference alkyd resin exhibited a char yield of 5% at 850 °C. The char yield at 850 °C increased by 7.5% and 13.1% for the resins with, respectively, 3 wt% P brought by PO_3_CR and PO_4_CR, respectively, compared to the reference resin. Those results were also in good agreement with the better char promotion effect of phosphate observed previously. Moreover, in the literature, most reports indicated that the char residue increases as the oxidation state of phosphorus increases [39,40,41,42]. In Scheme 4, a mechanism of the char formation in the condensed phase has been proposed. The char layer could be provided through cyclization, cross-linking and aromatization by dehydration of the polymeric structure, induced by phosphorus-cardanol. Moreover, each phosphorus-cardanol molecule contains three aromatic rings, which could bring high char content during combustion.

The residue increase may entail an insulating effect during combustion with a cone calorimeter. Indeed, during combustion, phosphonate and phosphate functions provide a continuous protective carbonaceous layer. This carbonaceous layer could insulate the polymer and prevent heat and gas transfer between gas and condensed phases [43,44]. Those results demonstrated that the replacement of harmful volatile compounds by environmentally friendly phosphorus reactive diluents provided excellent thermal stability.

**Figure 5:**
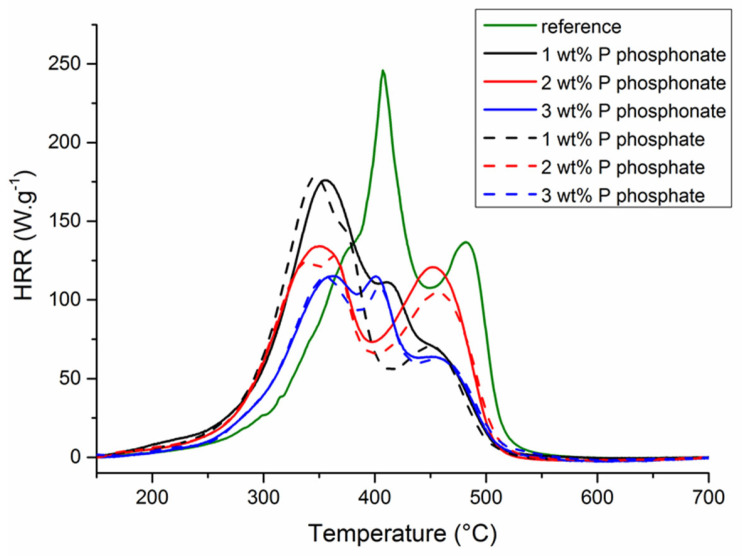
PCFC curves of reference alkyd resin and phosphorylated cardanol modified alkyd resins.

Table 4 summarizes the data obtained from PCFC analyses. In standard conditions, combustion is complete in the PCFC test due to the high combustor chamber (900 °C) in the presence of an excess of oxygen. The total heat release (THR) and the peak of heat release rate (pHRR) are important characteristics to evaluate the flame retardant (FR) properties of a material. Figure 5 exhibits the PCFC curves of the phosphorylated cardanol-modified alkyd resins. It can be noticed that all the alkyd resins exhibited two pHRR peaks. The increased amount of phosphorylated cardanol provided a reduction in pHRR and THR values which demonstrated the FR properties of PO_3_CR and PO_4_CR diluents. The pHRR of the PO_x_CR-free alkyd resin exhibited values of 246 and 137 W.g^−1^ whereas the pHRR were 176 and 110 W.g^−1^ for the alkyd resins with 1 wt% P of PO_3_CR and 178 and 71 W.g^−1^ for the alkyd resins with 1 wt% P of PO_4_CR. Moreover, the introduction of 3 wt% P of phosphorus-cardanol, decreased the pHRR to 115 W.g^−1^ and 114 or 108 W.g^−1^ for PO_3_CR and PO_4_CR, respectively.

The temperature of the pHRR decreased with the introduction of phosphorylated cardanol. Nevertheless, all the phosphorylated cardanol-modified alkyd resins exhibited a temperature of the pHRR around 350 °C. The growing amount of FR did not have an influence on this temperature.

THR values slightly decreased with increasing amounts of PO_x_CR diluent in alkyd resins. Indeed, the THR value was decreased from 25.7 KJ.g^−1^ for the PO_x_CR-free alkyd resin to 17.3 KJ.g^−1^ and 17.1 KJ.g^−1^ for the alkyd resins with 3 wt% P phosphonate-cardanol and phosphate-cardanol, respectively. Moreover, the energy of complete combustion (Δh) slightly decreased with the amount of phosphorylated cardanol.

Furthermore, the residual content increased from 0.5% to 12.0% and 15.5% for the reference resin and the resins with, respectively, 3 wt% P of PO_3_CR and PO_4_CR. These increases were consistent with the TGA results and demonstrated that phosphonate and phosphate compounds promoted char formation. Moreover, the residual content was slightly higher for the phosphate-cardanol, as already observed in TGA, which mainly acts in the condensed phase.

**Figure 6:**
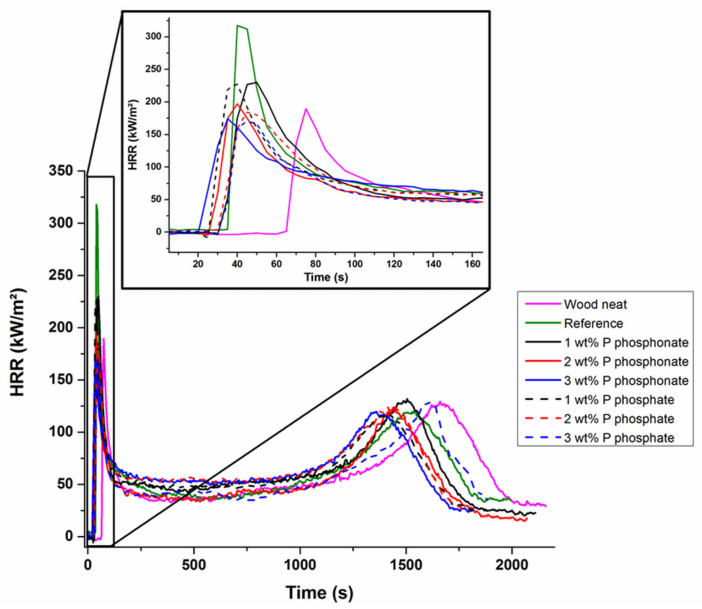
Curves of cone calorimeter tests for wood, reference alkyd resin and phosphorylated cardanol modified alkyd resins.

The cone calorimeter analyses provide important information on the fire behavior of a material [45]. Cone calorimeter tests were performed with a heat flux of 35 kW.m^−2^ under ventilated conditions. The results are summarized in Table 5, and Figure 6 exhibits the cone calorimeter curves. Tests were performed on wood samples with around 1 g of dry resin as a coating. The rate of heat release (HRR), as well as the total heat release (THR), are important parameters to characterize the fire performance of materials. The curves present two pHRR which is very common with wood samples. The first peak represents the heat release rate (pHRR_1_) reached just after the ignition. After this first peak corresponding to the decomposition of the top surface of wood including the coating, the heat release rate decreases until reaching a plateau which corresponds to the steady-state decomposition rate until reaching the bottom of the sample. The lower the plateau, the slower the pyrolysis front progresses through the thickness of the wood. When the heat reaches the unexposed surface (insulated by rockwool), the heat cannot be evacuated, leading to a second peak of heat release rate (pHRR_2_). This second peak of heat release rate is an artefact due to the geometry of the cone calorimeter test and is independent of the coating. All the samples have a wood thickness of around 25 ± 0.2 mm.

Wood without coating presented a moderate pHRR_1_ at 190 KW.m^−2^ and the FR-free alkyd resin led to a strong enhancement of the pHRR_1_ (up to 316 kW.m^−2^). Indeed, coatings usually negatively affect flame retardancy. The use of a coating layer usually negatively affects the flame retardancy of wood but provides protection from external aggressions such as UV, weather, and insects. Note that the typical effective heat of combustion of wood was close to 12 kJ.g^−1^ while the heat of combustion measured in PCFC was 25.8 kJ.g^−1^ for the FR-free alkyd resin. The objective was to provide a coating with better flame retardancy than a standard coating. The introduction of PO_3_CR and PO_4_CR diluents had a strong influence on the pHRR_1_ due to their ability to act in the condensed phase. Indeed, alkyd coatings with PO_x_CR released the energy slower due to the modification of the degradation mechanism due to the promoted char formation. Thereby, pHRR1 decreased with the amount of phosphorylated cardanol. The introduction of up to 3 wt% P phosphonate-cardanol has improved flame retardancy by decreasing the pHRR from 316 KW.m^−2^ to 174 KW.m^−2^. The lower pHRR for PO_4_CR was obtained with 3 wt% P and exhibited a value of 170 KW.m^−2^. Indeed, pHRR_1_ decreased by almost 46% compared to the PO_x_CR-free alkyd resins. Furthermore, the difference between PO_3_CR and PO_4_CR was not significant with the cone calorimeter results. The increased amounts of phosphorus content up to 2 wt% allowed to significantly improve the flame retardant properties. However, the change from 2 wt% P to 3 wt% P did not show a further improvement in flame retardancy. Moreover, the pHRR values for 2 and 3 wt% P were equivalent to the pHRR of the raw wood sample.

Time to ignition (TTI) measures the time to achieve sustained flaming combustion at a particular external heat flux. TTI was not impacted by the introduction of phosphorylated cardanol as all the alkyd resins provided a TTI of around 30 s. Nevertheless, TTI for all the woods with alkyd resins was at least two times lower than TTI for the uncoated wood. Indeed, the addition of alkyd coatings on the wood substrate considerably reduced the TTI.

The effective heat of combustion (EHC), the total heat release (THR) and the residual mass were not discussed because the coating only influenced pHRR_1_. All those results confirmed the benefits of phosphorus cardanol reactive diluent on flame retardant properties and the reduction in VOC amounts.

### 3.3. Film Properties

Films of the different resins have been applied with a film applicator to provide dry films with a final thickness of around 60 µm for all the alkyd resins. Adhesion, flexibility, gloss, drying time and chemical resistance have been determined and the results are presented in the following section in Table 6 and Table 7.

All the samples exhibited excellent adhesion properties. Indeed, the edges of the cuts were completely smooth and none of the squares of the grid were torn off. The results allow an evaluation as class 0 according to ISO2409:2020, except for the resin with 3 wt% P phosphate-cardanol which was rated at class 1. Indeed, small flakes of the coating were detached at intersections but less than 5% of the area was affected, which was not significant. The introduction of phosphorylated cardanol in alkyd resins did not impact the adhesion properties of the resins.

The hardness of the coating was impacted by the amount of phosphorylated cardanol introduced. The more flexible the film is, the faster the pendulum is damped and therefore the less oscillations are required (one second corresponds to one oscillation of the pendulum). Indeed, the more flexible is the film, the more it is able to absorb the energy of the pendulum. The number of oscillations decreased from 67 for the PO_x_CR-free-alkyd resin to 48 for the resins with 3 wt% P phosphorylated cardanol. The introduction of phosphorylated cardanol in alkyd resins provided more flexible films. Moreover, the flexibility increased with the amount of phosphorylated cardanol. These results agree with the DSC results which showed lower Tg values with the increase in phosphorylated cardanol amount in alkyd resins. Nevertheless, even if the films were more flexible with phosphorylated cardanol, the values were closed to that of the reference resin which means that the films remained hard enough allowing the resin to maintain its film-forming properties for wood substrates.

The gloss of the coatings was firstly measured at 60° to determine the gloss level. All the alkyd resins exhibited a high gloss level with values superior to 70 (between 95 and 98). The measurements were then studied at 20°. The reference resin showed a value of 81 and all the alkyd resins containing phosphorylated cardanol exhibited values between 79 and 84. The introduction of phosphorylated cardanol in alkyd resins did not impact the gloss of the resins.

The introduction of phosphorylated cardanol as a reactive diluent in alkyd resins increased the drying time. The drying time was increased by 1h30 min for the 1 wt% P modified alkyd resins compared to the drying time of PO_x_CR-free-alkyd resin. Moreover, the alkyd resins with 3 wt% P showed the longest drying time, which was 7 h. Those results are expected because the proportion of alkyd resins is lower in the formulations and the phosphorylated cardanol exhibited a low molecular weight and acted as a plasticizer by spacing the polymer chains. Thereby, more covalent bonds had to be formed to create a crosslinking network. Moreover, the incorporation of phosphorylated cardanol increased the content of fatty chains, allowing us to classify PO_x_CR-alkyd resins as medium oil length alkyd resins. The more an alkyd resin contains the same fatty chains, the longer the drying time [35]. Thereby, the drying time exhibited for the 1 wt% P and 2 wt% P modified alkyd resins remained competitive with a drying time of some commercial alkyd resins with medium/long oil length.

Table 7 presents the results of the determination of the chemical resistance of the resins. The higher the weight loss, the lower the residual mass percentage and the less resistant the resin is to the solvent. The chemical resistance of the alkyd resins in water was slightly better for PO_x_CR-modified alkyd resins. Indeed, all the PO_x_CR alkyd resins exhibited a residual mass percentage superior to 95% compared to the phosphorylated cardanol-free alkyd resin at 86%. The chemical resistance to HCl exposure presented a residual mass percentage above 90% for all alkyd resins containing PO_x_CR diluent versus 80% for the reference alkyd resin. In the case of the NaOH solution, the alkyd resin resistance was low compared to the other solutions. Indeed, all the alkyd resins exhibited a residual mass percentage of around 70%. Alkyd resins were highly resistant to NaCl solution with a residual mass superior to 95%. The introduction of phosphorylated cardanol showed higher chemical resistance for all the alkyd resins. Nevertheless, the lower resistance to alkali solution can be due to hydrolysable ester groups in the alkyd resins [46,47]. Indeed, saponification reactions could occur under those conditions.

## 4. Conclusions

New phosphorylated cardanol flame-retardants have been successfully synthesized and used as reactive diluents in alkyd resins in order to reduce the VOC content. Indeed, the viscosity of the alkyd resins has been strongly reduced. Phosphorylated cardanol compounds were grafted into alkyd resins after drying, highlighted by *GC* content and HRMAS NMR analysis. The introduction of phosphorylated cardanol reactive diluent slightly improved the chemical resistance of the alkyd resins. Nevertheless, as expected, the drying time was increased and the T_g_ was decreased with the amount of phosphorylated cardanol introduced but remained competitive. The fire behavior of those resins was investigated by TGA, PCFC and cone calorimeter. All the alkyd resins exhibited better thermal behavior with phosphorylated cardanol. It has been demonstrated that PO_3_CR and PO_4_CR provided very similar results, except for a better char yield with PO_4_CR. These results were expected as phosphate compounds mainly act in the condensed phase and promote char formation. The best result was obtained with an alkyd resin modified with 2 wt% P phosphate-cardanol. Indeed, this resin exhibited strong solvent reduction and high flame retardancy properties while maintaining adequate film properties for wood application.

## Figures and Tables

**Table 1:** Composition of phosphorylated cardanol reactive diluent alkyd resins at the same viscosity (5.3 Pa.s).

	Reference	Phosphonate			Phosphate		
**Wt% P**	0	1	2	3	1	2	3
**Xylene content (wt%)**	40	31	20	9	29	20	9
**Reactive diluent content (wt%)**	0	16	37	63	17	38	64
**Alkyd resin content (wt%)**	60	54	43	27	53	42	26
**N.V.C. (wt%)**	60	70	80	90	70	80	90

**Table 2:** Viscosity, Tg and gel content of phosphorylated cardanol reactive diluent alkyd resins with a fixed NVC at 60 wt%.

	Reference	Phosphonate			Phosphate		
**Wt% P**	0	1	2	3	1	2	3
**η (Pa.s) (xylene: 40 wt%)**	5.3	1.67	0.17	0.03	1.65	0.16	0.03
**Tg(°C)**	40	27	−3	−11	26	−3	−12
**Gel content (%)**	95	94	93	91	94	92	91

**Table 3:** Results of TGA analyses of PO_x_CR diluent alkyd resins.

	Wt% P	T_d,5wt%_ (°C)	T_d,50wt%_ (°C)	Residue at 850 °C (%)
**Reference**	0	140	350	5.0
**Phosphonate-cardanol**	Monomer	294	448	6.4
	1	180	368	7.5
	2	214	378	9.8
	3	221	390	12.5
**Phosphate-cardanol**	Monomer	350	462	16.5
	1	204	357	14.1
	2	242	367	15.2
	3	242	416	18.7

**Table 4:** Results of PCFC analyses of PO_x_CR diluent alkyd resins.

	Reference	Phosphonate			Phosphate		
**Wt% P**	0	1	2	3	1	2	3
**pHRR (W.g^−1^)**	246 137	176 110	134 120	115 114	178 71	125 105	115 108
**T at pHRR (°C)**	406 482	353 417	350 453	356 399	347 450	341 454	358 402
**THR (KJ.g^−1^)**	25.7	23.2	21.7	17.3	21.1	20.9	17.1
**Residue content (%)**	0.5	5.3	8.1	12.0	10.4	12.3	15.5
**Δ** **h (KJ.g^−1^)**	25.8	24.5	23.6	19.7	23.5	23.8	20.2

**Table 5:** Cone calorimeter analyses of phosphorylated cardanol reactive diluent alkyd resins.

	Wood Neat	Reference	Phosphonate			Phosphate		
**Wt% P**	0	0	1	2	3	1	2	3
**TTI (s)**	65	35	30	26	22	26	32	31
**pHRR_1_ (KW.m^−2^)**	190 ± 10	316 ± 11	229 ± 9	197 ± 10	174 ± 13	227 ± 11	186 ± 8	170 ± 7
**THR (KJ.g^−1^)**	9.4	10.0	10.0	8.7	8.9	8.6	8.8	8.4

**Table 6:** Adhesion, hardness, gloss and drying time of phosphorylated cardanol reactive diluent alkyd resins.

	Reference	Phosphonate			Phosphate		
**Wt% P**	0	1	2	3	1	2	3
**Adhesion**	0	0	0	0	0	0	1
**Hardness (s)**	67	58	48	48	67	58	48
**Gloss 20°/60°**	81/96	82/98	79/97	83/98	84/98	80/97	80/95
**Drying time (min)**	90	180	340	420	180	340	420

**Table 7:** Evaluation of chemical resistance of PO_x_CR-alkyd resins.

	Reference	Phosphonate			Phosphate		
**Wt% P**	0	1	2	3	1	2	3
**Water**	86 ± 2	96 ± 2	95± 3	95 ± 1	99 ± 1	98 ± 1	96 ± 2
**HCl (0.1 M)**	80 ± 4	91 ± 2	91 ± 1	92 ± 3	92 ± 1	91 ± 1	91 ± 1
**NaOH (0.1 M)**	69 ± 1	74 ± 2	75 ± 2	73 ± 3	74 ± 3	74 ± 2	73 ± 2
**NaCl (5%)**	95 ± 3	97 ± 1	96 ± 1	96 ± 1	95 ± 2	95 ± 4	97 ± 1

## Data Availability

Not applicable.

## Supplementary Materials

The following supporting information can be downloaded at: https://www.mdpi.com/article/10.3390/molecules27154880/s1. Figure S1: ^1^H NMR spectra of phosphonate-cardanol compound. Figure S2: ^1^H NMR spectra of phosphate-cardanol compound. Figure S3: ^1^H NMR spectrum of reference alkyd resin before curing. Figure S4: ^1^H NMR spectrum of phosphate cardanol modified alkyd resin (1 wt% P) before curing. Figure S5: ^1^H NMR spectrum of phosphate cardanol modified alkyd resin (2 wt% P) before curing. Figure S6: ^1^H NMR spectrum of phosphate cardanol modified alkyd resin (3 wt% P) before curing. Figure S7: ^1^H HRMAS NMR spectrum of reference alkyd resin after curing. Figure S8: ^1^H HRMAS NMR spectrum of phosphate cardanol modified alkyd resin (1 wt% P) after curing. Figure S9: ^1^H HRMAS NMR spectrum of phosphate cardanol modified alkyd resin (2 wt% P) after curing. Figure S10: ^1^H HRMAS NMR spectrum of phosphate cardanol modified alkyd resin (3 wt% P) after curing. Figure S11: Viscosity of the alkyd resins at different amounts of phosphorus-cardanol reactive diluents. Figure S12: DSC curves of alkyl resins. Table S1: Cutting tool blades depending on the coating thickness. Table S2: Damages depending on the ISO 2409 class.

## Sample Availability

Samples of the compounds are available from the authors.

## References

[1] Hofland A. (2012). Alkyd Resins: From down and out to Alive and Kicking. Prog. Org. Coat..

[2] La Nasa J., Degano I., Modugno F., Colombini M.P. (2013). Alkyd Paints in Art: Characterization Using Integrated Mass Spectrometry. Anal. Chim. Acta.

[3] Wicks Z.W. (2000). Alkyd Resins. Kirk-Othmer Encycl. Chem. Technol..

[4] Elliott W.T. (1993). Alkyd Resins. Surface Coatings.

[5] Assanvo E.F., Gogoi P., Dolui S.K., Baruah S.D. (2015). Synthesis, Characterization, and Performance Characteristics of Alkyd Resins Based on Ricinodendron Heudelotii Oil and Their Blending with Epoxy Resins. Ind. Crops Prod..

[6] Chiplunkar P.P., Pratap A.P. (2016). Utilization of Sunflower Acid Oil for Synthesis of Alkyd Resin. Prog. Org. Coat..

[7] Chardon F., Denis M., Negrell C., Caillol S. (2021). Hybrid Alkyds, the Glowing Route to Reach Cutting-Edge Properties?. Prog. Org. Coat..

[8] Nabuurs T. (1997). Alkyd-Acrylic Composite Emulsions Polymerization and Morphology.

[9] Ling J.S., Ahmed Mohammed I., Ghazali A., Khairuddean M. (2014). Novel Poly(Alkyd-Urethane)s from Vegetable Oils: Synthesis and Properties. Ind. Crops Prod..

[10] Nalawade P.P., Soucek M.D. (2015). Modified Soybean Oil as a Reactive Diluent: Coating Performance. J. Coat. Technol. Res..

[11] Overbeek A. (2010). Polymer Heterogeneity in Waterborne Coatings. J. Coat. Technol. Research.

[12] Sharmin E., Zafar F., Akram D., Alam M., Ahmad S. (2015). Recent Advances in Vegetable Oils Based Environment Friendly Coatings: A Review. Ind. Crops Prod..

[13] Wang H., Zhang C., Zhou Y., Zhou Q. (2020). Improvement of Corrosion Resistance and Solid Content of Zinc Phosphate Pigmented Alkyd Coating by Methacrylated Cardanol. Mater. Today Commun..

[14] Athawale V.D., Nimbalkar R.V. (2011). Waterborne Coatings Based on Renewable Oil Resources: An Overview. JAOCS. J. Am. Oil Chem. Soc..

[15] Wang H., Hu G., Liu X., Guo L., Li X., Guo R., Li Y. (2022). Concurrent Alkylation and Crosslinking of Polyaniline for Enhanced Anticorrosive Performance of Waterborne Alkyd Coating. Prog. Org. Coat..

[16] Njuku F.W., Mwangi P.M., Thiong’o G. (2014). Evaluation of Cardanol Acetate as a Reactive Diluent for Alkyd Coatings. Int. J. Adv. Res..

[17] Jagtap A.R., More A. (2021). Developments in Reactive Diluents: A Review. Polym. Bull..

[18] Jones F.N. (2012). Alkyd Resins. Ullman’s Ecyclopedia Ind. Chem..

[19] Bora M.M., Deka R., Ahmed N., Kakati D.K. (2014). Karanja (*Millettia pinnata* (L.) Panigrahi) Seed Oil as a Renewable Raw Material for the Synthesis of Alkyd Resin. Ind. Crops Prod..

[20] Lee R., Gryn’ova G., Ingold K.U., Coote M.L. (2016). Why Are Sec-Alkylperoxyl Bimolecular Self-Reactions Orders of Magnitude Faster than the Analogous Reactions of Tert-Alkylperoxyls? The Unanticipated Role of CH Hydrogen Bond Donation. R. Soc. Chem..

[21] Honzíček J. (2019). Curing of Air-Drying Paints: A Critical Review. Ind. Eng. Chem. Res..

[22] Li W.S.J., Cuminet F., Ladmiral V., Lacroix-Desmazes P., Caillol S., Negrell C. (2021). Phosphonated and Methacrylated Biobased Cardanol Monomer: Synthesis, Characterization and Application. Prog. Org. Coat..

[23] Ionescu M., Wan X., Bilić N., Petrović Z.S. (2012). Polyols and Rigid Polyurethane Foams from Cashew Nut Shell Liquid. J. Polym. Environ..

[24] Pillai C.K.S., Prasad V.S., Sudha J.D., Bera S.C., Menon A.R. (1990). Polymeric Resins from Renewable Resources. II Synthesis and Characterization of Flame- Retardant Prepolymers from Cardanol. J. Appl. Polym. Sci..

[25] Wang H., Zhang C., Zeng W., Zhou Q. (2019). Making Alkyd Greener: Modified Cardanol as Bio-Based Reactive Diluents for Alkyd Coating. Prog. Org. Coat..

[26] Hobbs C.E. (2019). Recent Advances in Bio-Based Flame Retardant Additives for Synthetic Polymeric Materials. Polymers.

[27] Ecochard Y., Decostanzi M., Negrell C., Sonnier R., Caillol S. (2019). Cardanol and Eugenol Based Flame Retardant Epoxy Monomers for Thermostable Networks. Molecules.

[28] Phalak G., Patil D., Patil A., Mhaske S. (2019). Synthesis of acrylated cardanol diphenyl phosphate for UV curable flame-retardant coating application. Eur. Polym. J..

[29] Mestry S., Kakatkar R., Mhaske S.T. (2019). Cardanol derived P and Si based precursors to develop flame retardant PU coating. Prog. Org. Coat..

[30] Laoutid F., Bonnaud L., Alexandre M., Lopez-Cuesta J.M., Dubois P. (2009). New Prospects in Flame Retardant Polymer Materials: From Fundamentals to Nanocomposites. Mater. Sci. Eng. R Rep..

[31] Huggett C. (1980). Estimation of Rate of Heat Release by Means of Oxygen Consumption Measurements. Fire Mater..

[32] Williamson W. (1852). On Etherification. Q. J. Chem. Soc. Lond..

[33] Ménard R., Negrell-Guirao C., Ferry L., Sonnier R., David G. (2014). Synthesis of Biobased Phosphate Flame Retardants. Pure Appl. Chem..

[34] Illy N., Fache M., Ménard R., Negrell C., Caillol S., David G. (2015). Phosphorylation of Bio-Based Compounds: The State of the Art. Polym. Chem..

[35] Mustafa S.F.M., Gan S.N., Yahya R. (2013). Synthesis and Characterization of Novel Alkyds Derived from Palm Oil Based Polyester Resin. Asian J. Chem..

[36] Waitara F.N. (2014). Evaluation of Cashew Nut Shell Liquid Based Products as Reactive Diluents for Alkyd Coatings. Ph.D. Thesis.

[37] Green J. (1996). A Review of Phosphorus-Containing Flame Retardants. J. Fire Sci..

[38] Fang F., Huo S., Shen H., Ran S., Wang H., Song P., Fang Z. (2020). A bio-based ionic complex with different oxidation states of phosphorus for reducing flammability and smoke release of epoxy resins. Compos. Commun..

[39] Schartel B. (2010). Phosphorus-based flame retardancy mechanisms-old hat or a starting point for future development?. Materials.

[40] Braun U., Balabanovich A.I., Schartel B., Knoll U., Artner J., Ciesielski M., Döring M., Perez R., Sandler J.K.W., Altstädt V. (2006). Influence of the oxidation state of phosphorus on the decomposition and fire behaviour of flame-retarded epoxy resin composites. Polymer.

[41] Velencoso M.M., Battig A., Markwart J.C., Schartel B., Wurm F.R. (2018). Molecular Firefighting—How Modern Phosphorus Chemistry Can Help Solve the Flame Retardancy. Angew. Chem. Int. Ed..

[42] Price D., Horrocks A.R. (2009). Fire Retardancy of Polymeric Materials.

[43] Qi J., Wen Q., Zhu W. (2018). Research Progress on Flame-Retarded Silicone Rubber. IOP Conf. Ser. Mater. Sci. Eng..

[44] Hamdani S., Longuet C., Perrin D., Lopez-cuesta J.M., Ganachaud F. (2009). Flame Retardancy of Silicone-Based Materials. Polym. Degrad. Stab..

[45] McNally T., Pötschke P., Halley P., Murphy M., Martin D., Bell S.E.J., Brennan G.P., Bein D., Lemoine P., Quinn J.P. (2005). Polyethylene Multiwalled Carbon Nanotube Composites. Polymer.

[46] Islam M.R., Beg M.D.H., Jamari S.S. (2014). Alkyd Based Resin from Non-Drying Oil. Procedia Eng..

[47] Ikhuoria E.U., Aigbodion A.I., Okieimen F.E. (2003). Enhancing the Quality of Alkyd Resins Using Methyl Esters of Rubber Seed Oil. Trop. J. Pharm. Res..

