# Supplementary material for: Phosphorus Modified Cardanol: A Greener Route to Reduce VolaTile Organic Compounds and Impart Flame Retardant Properties to Alkyd Resin Coatings
Source: Molecules. 2022 Jul 30;27(15):4880. doi: 10.3390/molecules27154880 (PMC9369946; doi:10.3390/molecules27154880)
Supplement: Supplementary file 1 [file molecules-27-04880-s001.zip › molecules-1819558-supplementary.pdf]

Phosphorus modified cardanol: a greener route to reduce volatile organic compounds and impart flame retardant properties to alkyd resins coatings.

Maxinne Denis[a,b], Damien Le Borgne[b], , Rodolphe Sonnier[c], Sylvain Caillol[a] , Cédric Totee[a], Claire Negrell[a*]

[a] ICGM, Univ Montpellier, CNRS, ENSCM, Montpellier, France
[b] Lixol, groupe Berkem, 20 Rue Jean Duvert, 33290 Blanquefort, France[2]
[c] Polymers Composites and Hybrids (PCH), IMT Mines Ales, Ales, France
* *Corresponding author: Claire Negrell, email:* Claire.negrell@enscm.fr

1. H NMR spectra

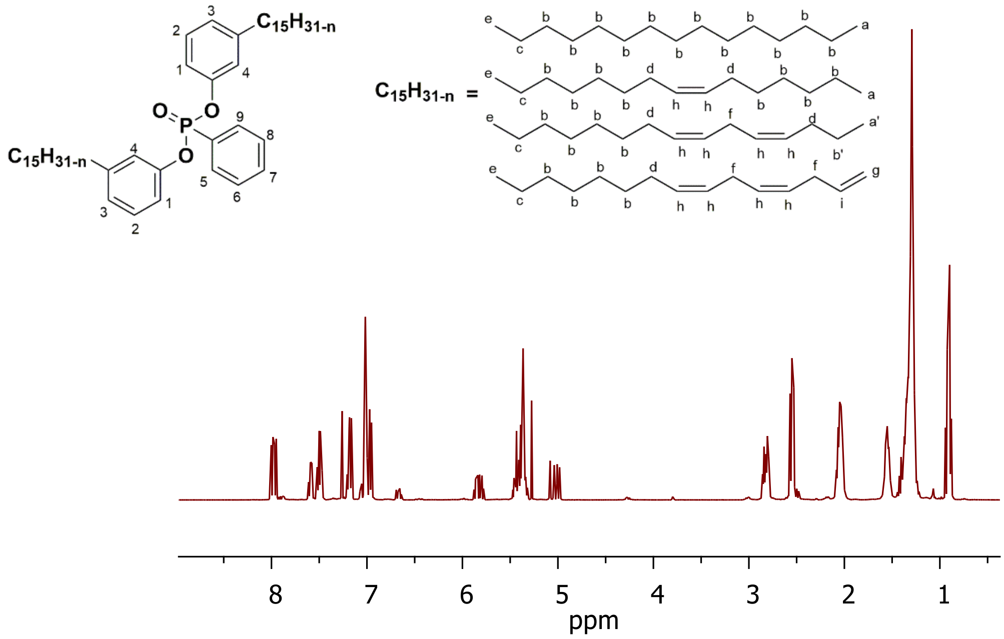

Figure S1. ^{1}H NMR spectra of phosphonate-cardanol compound.

^{1}H NMR (400 MHz, $CDCl_3$, ppm): δ = 7.99 (m, 2H, aromatic protons 5 and 9), 7.59 (dt, 1H, aromatic protons 7), 7.50 (m, 2H aromatic protons 6 and 8), 7.19 (t, 2H, aromatic protons 2), 6.99 (m, 6H, aromatic protons 1, 3, and 4), 5.80–5.90 (m, 1H, CH of C_{15} chain, proton i), 5.34–5.50 (m, CH of C_{15} chain, internal double bond protons h), 4.98–5.11 (m, 2H, CH_2 of C_{15} chain, protons of the terminal double bond g), 2.82 (m, 2H, CH_2 of C_{15} chain, protons f), 2.61 (t, 2H, CH_2 of C_{15} chain, protons e), 2.00–2.15 (m, 2H, CH_2 of C_{15} chain, protons d), 1.64 (m, 2H, CH_2 of C_{15} chain, protons c), 1.20–1.50 (m, CH_2 of C_{15} chain, protons b, b′), 0.92 (m, 3H, CH_3 of C_{15} chain, terminal –CH_3, protons a, a′).

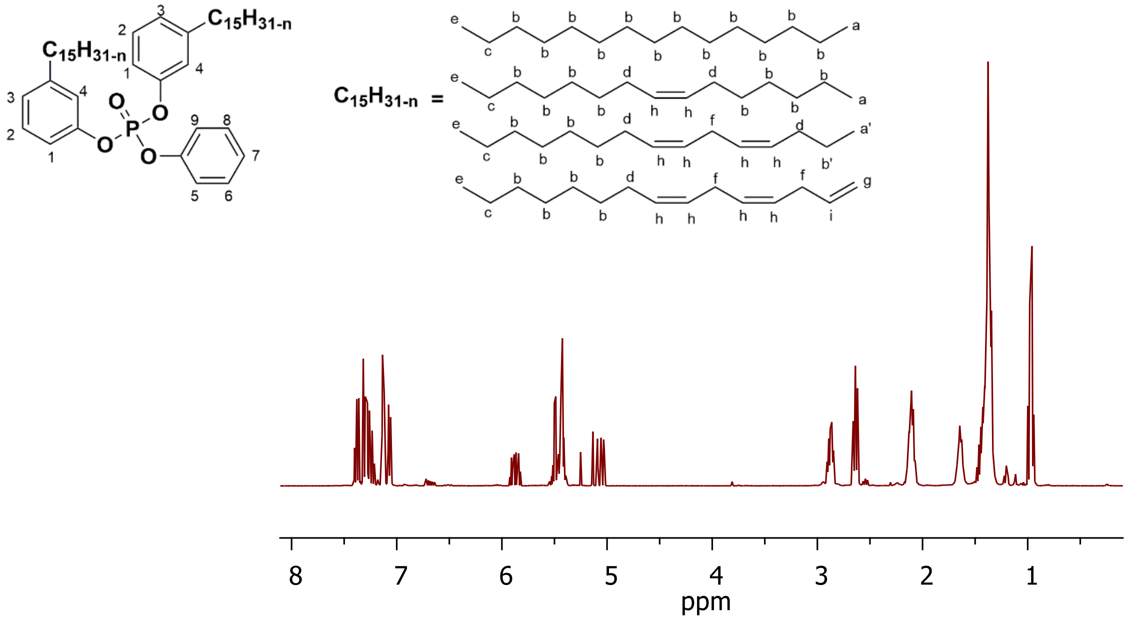

Figure S2. ^{1}H NMR spectra of phosphate-cardanol compound.

^{1}H NMR (400 MHz, $CDCl_3$, ppm): δ: 7.37 (m, 2H, aromatic protons 5 and 9), 7.24-7.28 (m, 3H, aromatic protons 6, 7 and 8), 7.19-7.24 (m, 2H, aromatic proton 2), 7.05 (m, 6H, aromatic protons 1, 3, and 4), 5.80–5.90 (m, 1H, CH of C_{15} chain, proton i), 5.34–5.50 (m, CH of C_{15} chain, internal double bond protons h), 4.98–5.11 (m, 2H, CH_2 of C_{15} chain, protons of the terminal double bond g), 2.82 (m, 2H, CH_2 of C_{15} chain, protons f), 2.61 (t, 2H, CH_2 of C_{15} chain, protons e), 2.00–2.15 (m, 2H, CH_2 of C_{15} chain, protons d), 1.64 (m, 2H, CH_2 of C_{15} chain, protons c), 1.20–1.50 (m, CH_2 of C_{15} chain, protons b, b′), 0.92 (m, 3H, CH_3 of C_{15} chain, terminal –CH_3, protons a, a′).

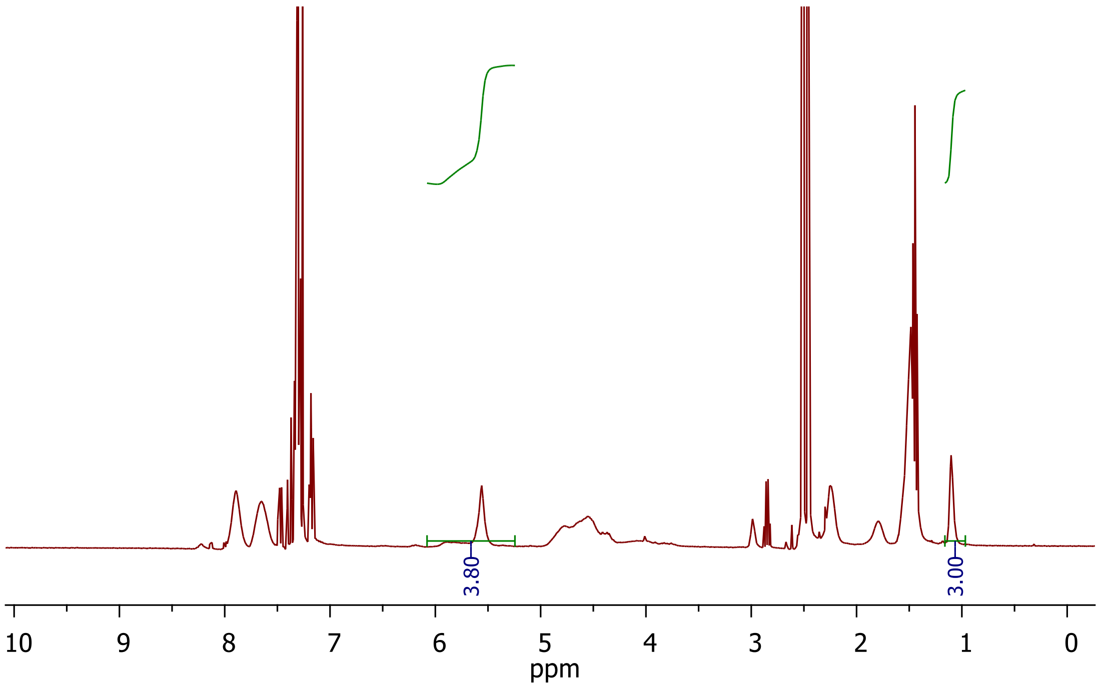

Figure S3. ^{1}H NMR spectrum of reference alkyd resin before curing.

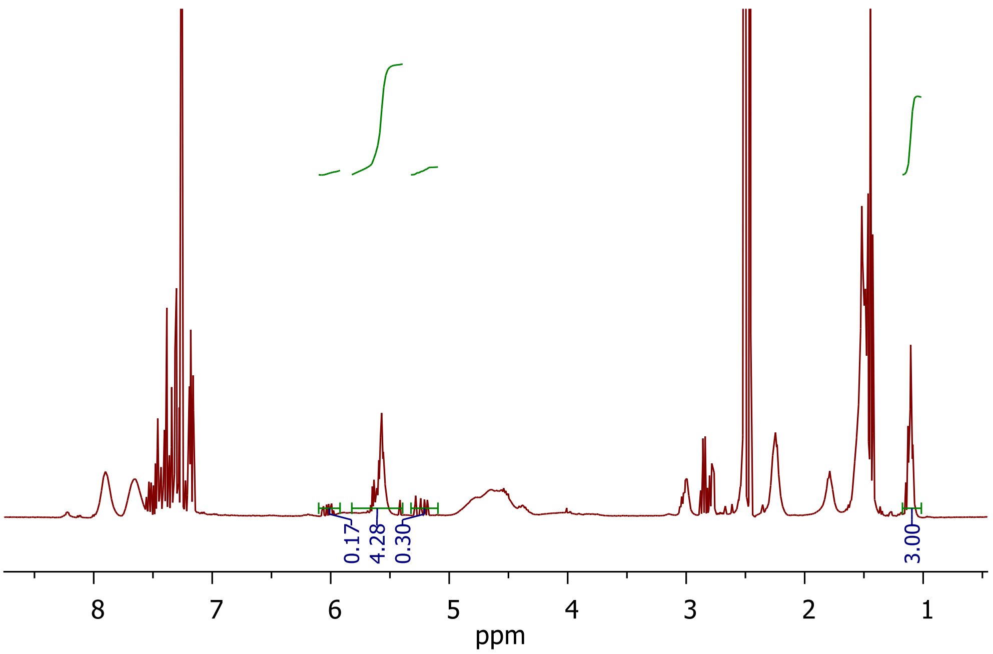

Figure S4. ^{1}H NMR spectrum of phosphate cardanol modified alkyd resin (1 wt% P) before curing.

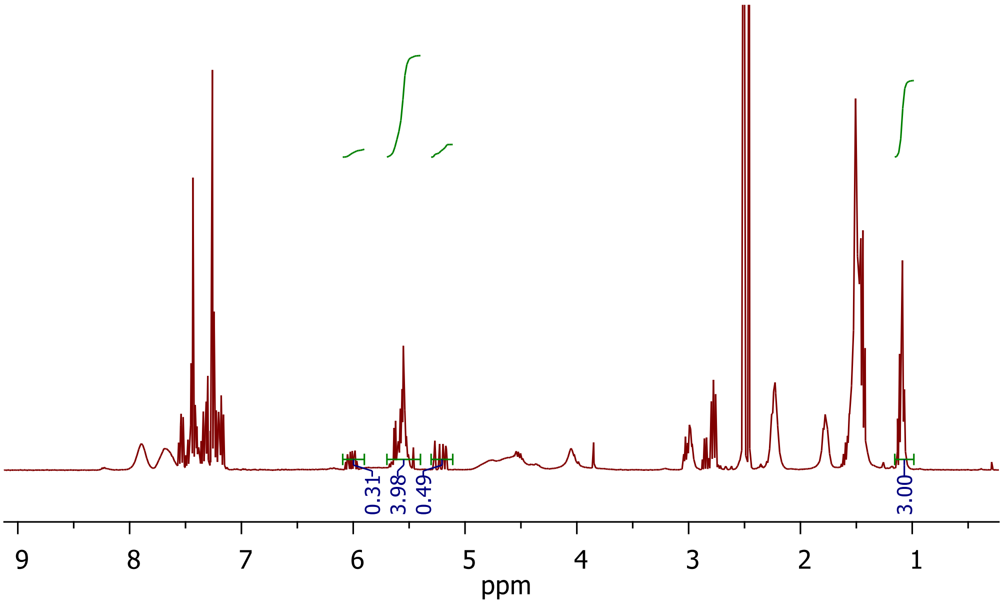

Figure S5. ^{1}H NMR spectrum of phosphate cardanol modified alkyd resin (2 wt% P) before curing.

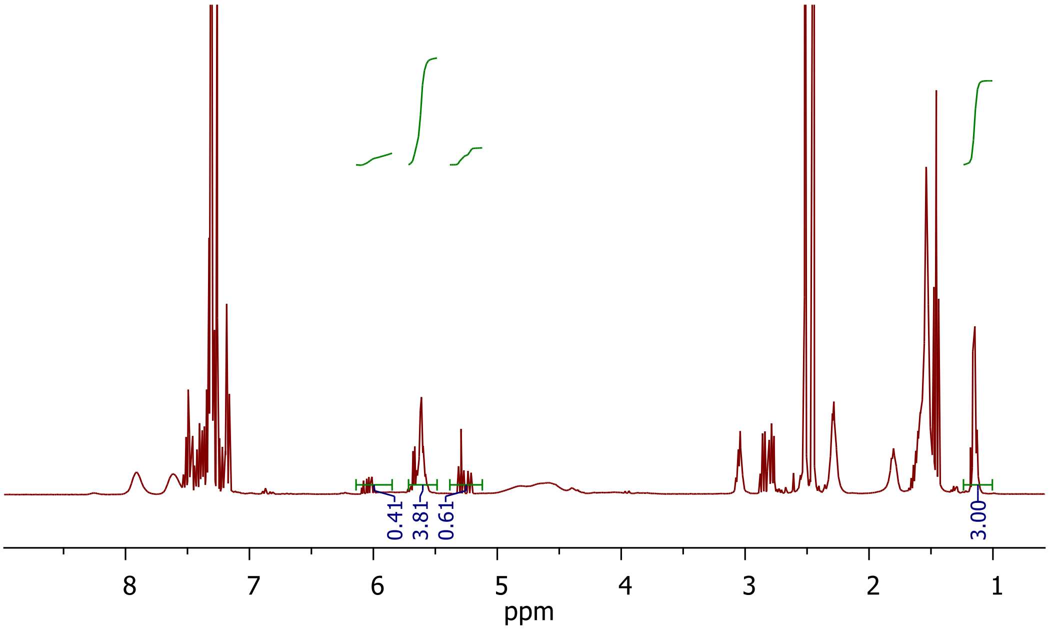

Figure S6. ^{1}H NMR spectrum of phosphate cardanol modified alkyd resin (3 wt% P) before curing.

2. HRMAS spectra

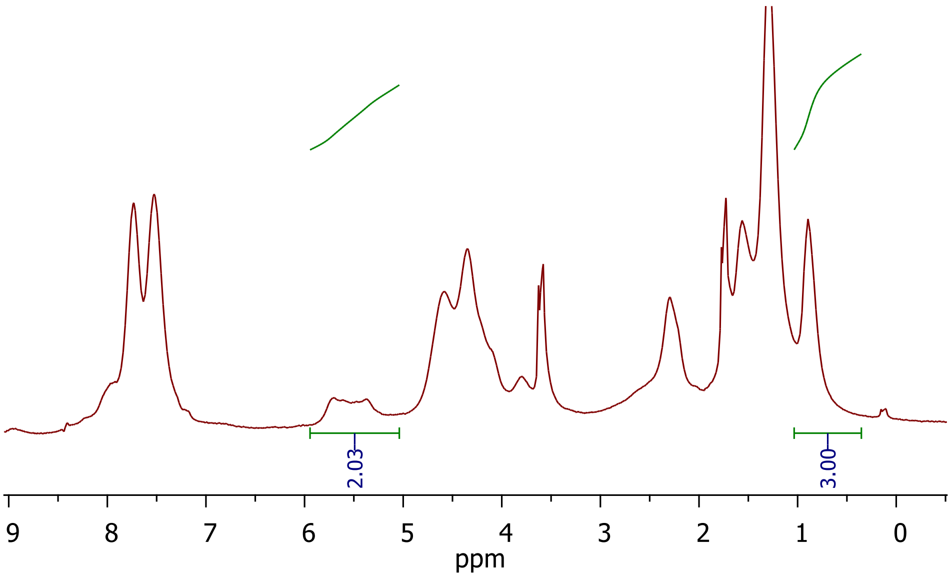

Figure S7. ^{1}H HRMAS NMR spectrum of reference alkyd resin after curing.

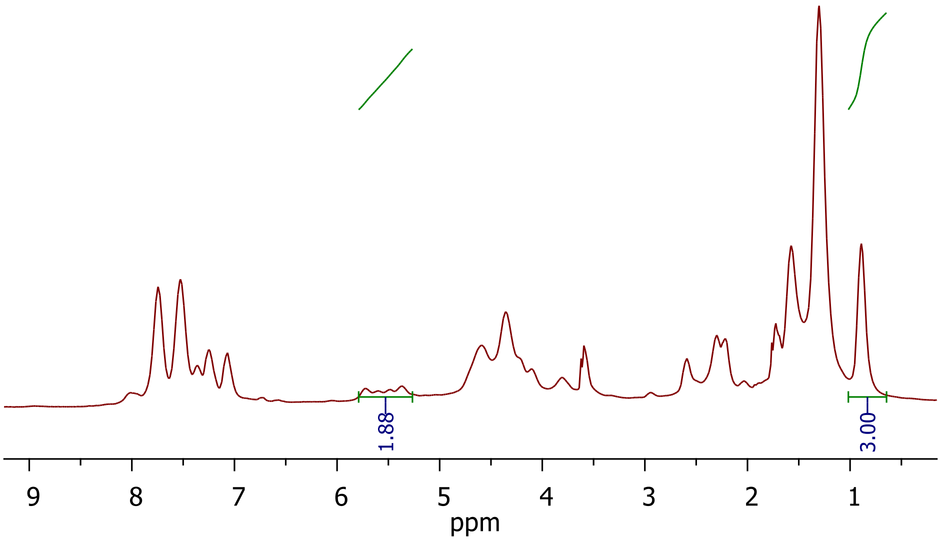

Figure S8. ^{1}H HRMAS NMR spectrum of phosphate cardanol modified alkyd resin (1 wt% P) after curing.

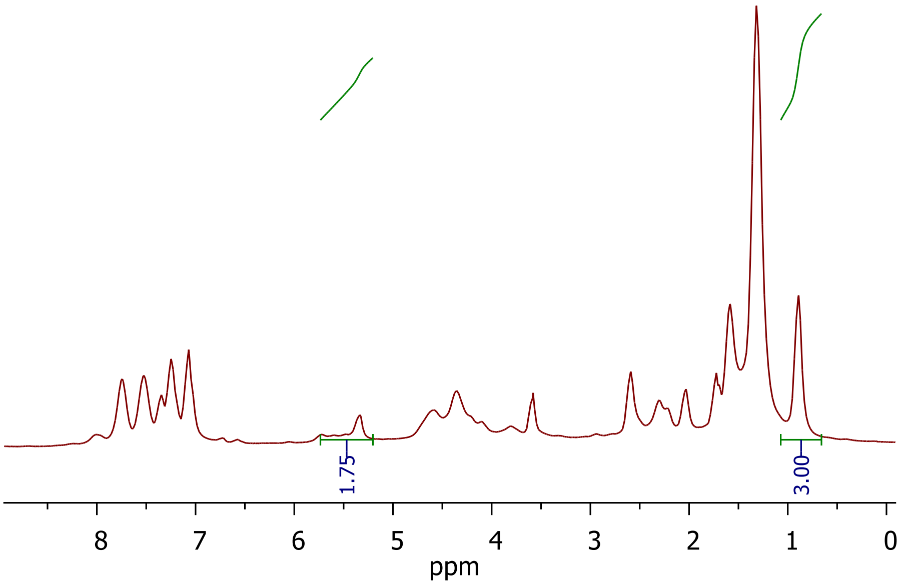

Figure S9. ^{1}H HRMAS NMR spectrum of phosphate cardanol modified alkyd resin (2 wt% P) after curing.

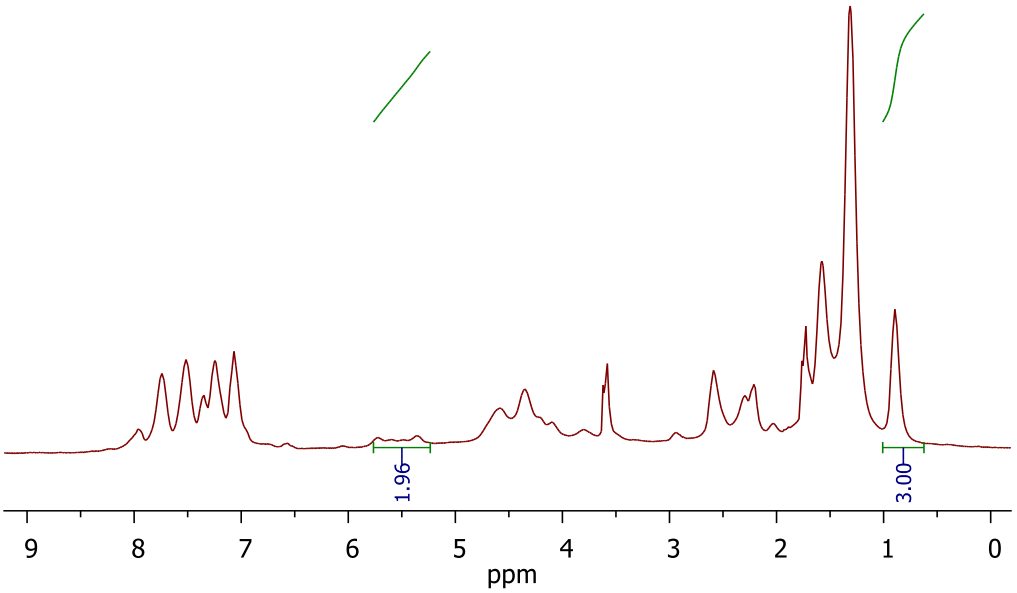

Figure S10. ^{1}H HRMAS NMR spectrum of phosphate cardanol modified alkyd resin (3 wt% P) after curing.

3. Viscosity

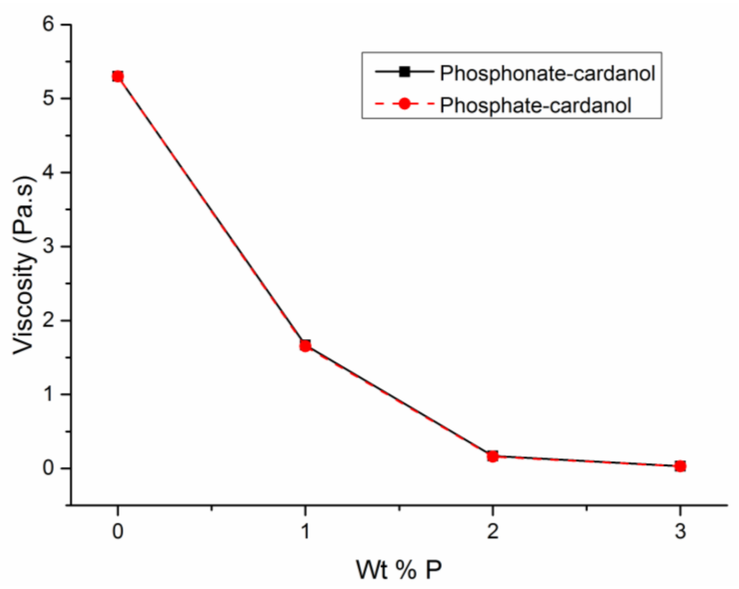

Figure S11. Viscosity of the alkyd resins at different amounts of phosphorus-cardanol reactive diluents.

4. DSC

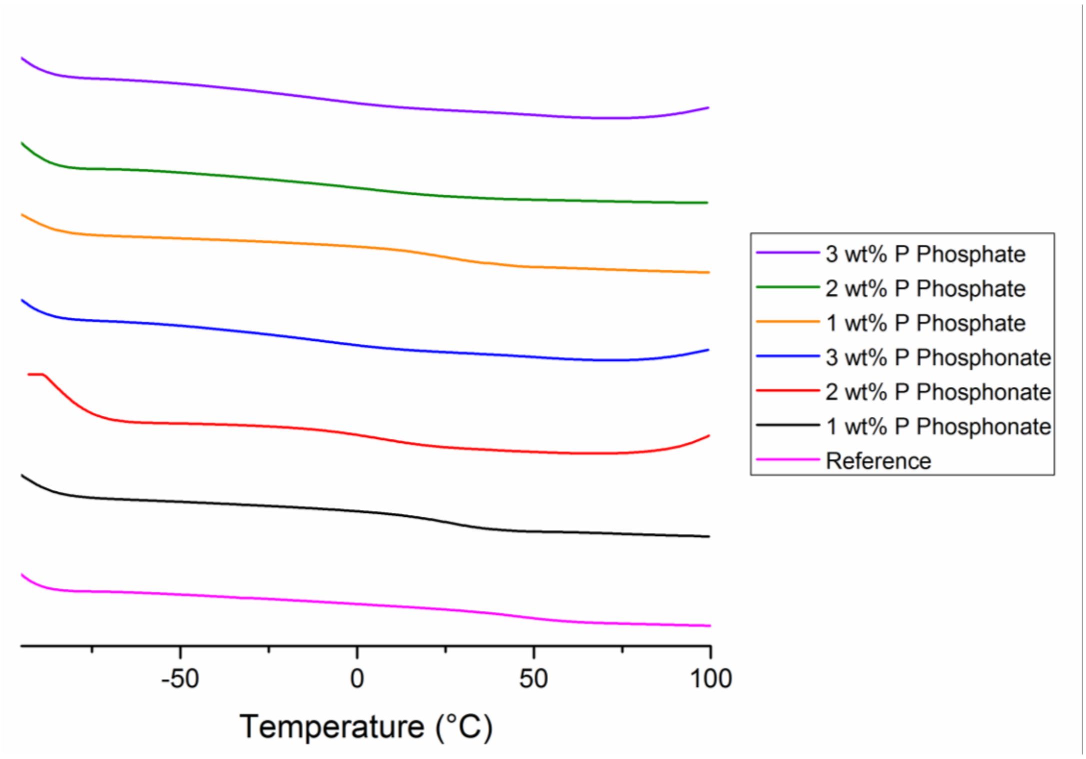

Figure S12. DSC curves of alkyd resins.

5. Standards

5.1. ISO 2409: adhesion test

This standard is used to determine the resistance of coating to be separated from its support. A grid is cut through the coating by incisions which reach the surface of the support. Depending on the coating thickness, the space between the cutting tool blades are different and they are listed in Table S1.

Table S1. Cutting tool blades depending on the coating thickness.

Coating thickness	*Space between the blades*
0 - 60 µm	1 mm
60 - 120 µm	2 mm
>120 µm	3 mm

A grid is obtained by using the appropriate cutting tool blades on the coating. Then, a scotch tape is stuck and removed five times. The grid is examined and depending on the damages a class from 0 to 5 is given (Table S2).

Table S2. Damages depending on the ISO 2409 class.

ISO 2409 class	*ASTMD3359 class*	*Pictures of damages*	*Evaluation criteria*
0	5B	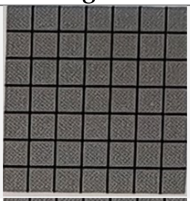	Edges of the cuts are completely smooth; none of the squares of the grid is detached.
1	4B	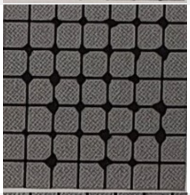	Small flakes of the coating are detached at intersections; less than 5 % of the area is affected.
2	3B	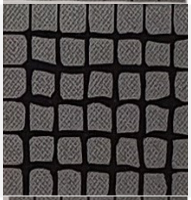	Small flakes of the coating are detached along edges and at intersections of cuts. The area affected is 5 to 15 % of the grid.
3	2B	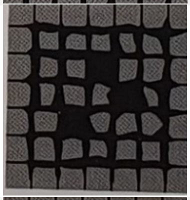	The coating has flakes along the edges and on parts of the squares. The area affected is 15 to 35 % of the grid.
4	1B	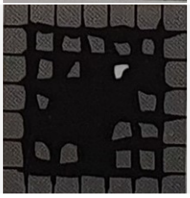	The coating has flaked along the edges of cuts in large ribbons and whole squares have detaches. The area affected is 35 to 65 % of the grid.
5	0B		The area affected is more than 65 % of the grid.

5.2. ISO 1522: hardness

The ISO 1522 is used to determine the damping of a pendulum on a coating. The test consists of measuring the damping time (or number of oscillations before damping) of a pendulum resting on the coating by mean on two steal ball. The pendulum is spread at an angle 12 ° from vertical, then released. The pendulum is considered to be damped when the maximum angle amplitude of the pendulum is less than or equal to 4 °. One oscillation corresponds to one second.

5.3. ISO 2813: gloss

The ISO 2813 (similar to ASTM D 523) is used to determine the gloss of a coating according to a determined measurement angle. Firstly, the gloss level is determined with a measurement at 60 °. If the value is inferior to 10 gloss unit (GU), the coating is low gloss/matt and the measurement should be made at 85 °; if the value is included between 10 to 70 GU, then the coating is medium gloss. Finally, if the value is superior to 70 GU, the coating is highly gloss and the measurement should be made at 20 °.
